# Identity Transfer and Identity Restoration in Facial Allotransplantation

**Published:** 2011-04-29

**Authors:** Ajay Modgil

**Affiliations:** Home Clinic, Vikas Nagar, Una 174303, Himachal Pradesh, India

## Abstract

**Objective:** Facial allotransplantation is fast becoming a reconstructive option for severely disfigured individuals, but it is still in experimental stage. Facial allotransplantation will be considered fully ethical only when it addresses the recipient's identity concerns. There exist no such studies that quantify and predict identity changes following change in geometry of underlying facial skeleton and overlying soft tissue morphology. I objectively address these identity concerns in the present study. **Methods:** Using software (Mimics version 14.0, Materialise, Plymouth, Michigan), I developed 3-dimensional facial models from the computed tomographic images of 4 identical and 1 nonidentical twin pairs. On the basis of cranial base anatomy, 3-dimensional coordinate values of selected landmark locations were measured (Surgicase, Materialise, Plymouth, Michigan) and a morphometric method was applied to quantify identity differences between them. Identity parameters were drawn on a diagrammatic chart that depicted the biophysical identity range. Using software (Mimics version 14.0), facial allotransplant simulation was done involving most of the nasal bone, maxilla, and zygoma. Morphological parameters of resulting new identity were drawn on diagrammatic chart and if they fell within the chart, facial allotransplantation resulted in identity transfer. Moreover, a scale was used to quantify identity transfer and identity restoration resulting from facial allotransplantation. **Results:** Identity changes of facial allotransplantation are objectively quantified that can be used as a communication tool in consent taking process. **Conclusion:** My findings suggest that donor facial allograft may be identified on the recipient's face depending upon underlying craniofacial morphology and accordingly, identity changes can be suitably predicted.

The first radical facial surgery that restored facial appearance was done in India in 1994, when Dr Thomas, a microsurgeon, replanted the entire face of a 9-year-old girl, who had lost her face and scalp in a threshing machine accident.^1^ This might have inspired plastic surgeons over the world to wonder if new faces could be transplanted onto severely disfigured patients. After much ethical debate, first human facial allotransplantation was done in Lyon, France, in 2005.^2^ Facial allotransplantation prompts immediate aesthetic concern as to whose identity the recipient will finally assume after this innovative surgery. Moreover, facial allotransplantation will be considered fully ethical only when informed consent process addresses the identity concerns to potential recipients and donors.^3^

Few authors have investigated the identity issues of facial allotransplantation. Siemionow and Agaoglu^4^ found that final appearance of a recipient is a mixture of features of both recipient and donor. Baccarani et al^5^ concluded that recipient's appearance depends upon bony framework of the underlying cranium. Pomahac et al^6^ found negligible appearance transfer to the recipients from donors.

However, no study quantifies and predicts identity changes accompanying altered morphology of overlying soft tissues and geometry of underlying bony framework. Only 9 face transplants have been done around the world,^7^ and experimental nature of facial allotransplantation itself is a limiting factor in decision-making with regard to donation and receipt of facial grafts. A method is urgently required that not only predicts and quantifies the identity changes but also can be used as a scientific tool to educate potential recipients and donors to advance the noble cause of facial allotransplantation.

The aim of the study was to

1. predict and quantify identity changes of facial allotransplantation and
2. predict identity changes relative to recipient's family facial phenotype.

## METHODS AND MATERIALS

### Subjects

Five twins—aged between 20 and 33 years (mean age, 23)—were selected on the basis of lack of any craniofacial trauma, surgery, congenital deformity, or unknown lesion and the fact that they were not being treated for any medical deformity, underwent computed tomographic (CT) scan. All twins were of north Indian descent that had symmetrical appearances and had been living together for most of their lifetime. Four twin pairs were monozygotic and 1 twin pair was dizygotic as was determined at the time of their birth on the basis of anthropometric and serologic methods. They were explained in details about the purpose and nature of study in their native language before informed consent was taken.

### Imaging and software

Computed tomographic (multislice) images, with a high-resolution bone algorithm (512 × 512 matrix, 120 KV, and 200 mA), were taken using CT scanner (Siemens, Germany), and image covered the area from vertex to submandibular region of craniofacial complex. Acquired CT slice data were imported to workstation (Windows 7, Intel i5-650 dual-core processor, 512 MB NVIDIA GeForce graphics card) and reconstructed in 3 dimensions. A life size facial model was developed using segmentation and volume rendering techniques (Mimics version 14.0, Materialise, Michigan, USA). Hard and soft tissues were separated by threshold-based segmentation, based on differences in their permeability to x-rays.

### Three-dimensional morphometric analyses

Software (Surgicase, Materialise, Plymouth, Michigan) allowed creating a template of landmark locations, (Table 1) and measurements were recorded between them. Three-dimensional (3D) spatial coordinate system was defined by using 4 landmarks: nasion, basion, prosthion, and sella. The y-z plane passed through nasion, basion, and prosthion in midline, x-y plane was perpendicular to y-z plane, passing through nasion and sella while z-x plane was perpendicular to y-z and x-y planes, passing through basion.

**Figure 1:**
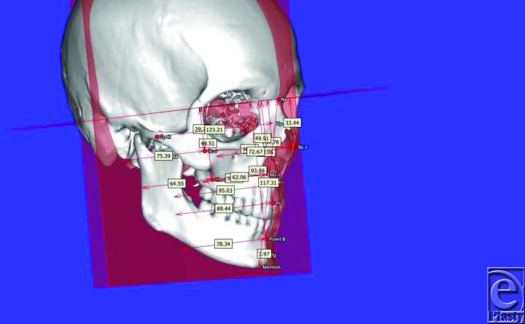
Landmark locations and their 3D measurements relative to set planes.

**Figure 2:**
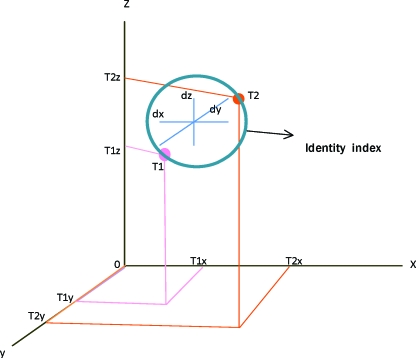
Identity Index was calculated as displacement vector (difference between 3-dimensional coordinates (T1x-, T1y-, T1z- and T2x-, T2y-, T2z-) of particular landmark location of a twin pair, T1 and T2).

X-axis was determined roughly parallel to right-left direction of subject's maxillofacial skeleton. Y-axis and z-axis corresponded to anterior-posterior and inferior-superior directions, respectively. Three-dimensional coordinate values (dx, dy, dz) were calculated for landmarks Bc, Pr, and Mx with origin (0, 0, 0) set at Ba. For Pr, only y- and z-axis values while for bilateral landmarks, x-axis (horizontal difference between 2 landmarks), y- and z-axis coordinate values (average of right and left side values) were measured (Fig 1). Displacement vector was calculated as the difference between 3D coordinate values of a twin pair (T1, T2) and was named as identity index for that particular landmark location (Fig 2).

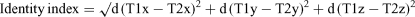



**Figure 3:**
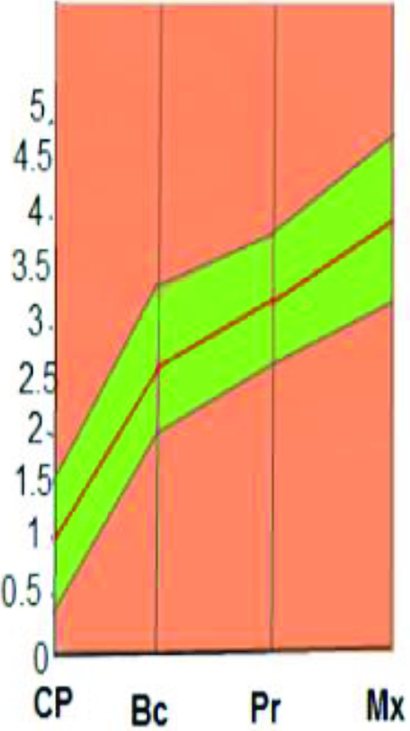
Diagrammatic chart indicates biophysical identity range (green zone) of landmark locations. Red line in the middle indicates mean values of identity indices (mm) while dark lines indicate values of one standard deviation on either side.

Mean and standard deviation values of identity indices (mm) and chin protuberance difference (mm) of identical twins were drawn on a diagrammatic chart that indicated the biophysical identity range (Fig 3).

### Family morphometric analyses

**Figure 4:**
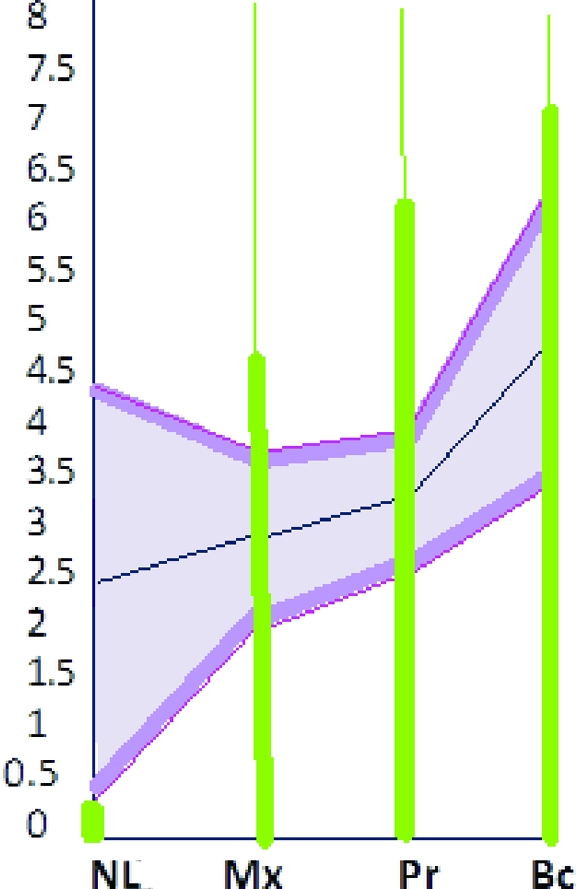
A diagrammatic chart indicates the family identity pool. Family identity indices (mm) of recipient were plotted to find if recipient's new identity became part of family phenotype.

It has 3 parts:

1. Four subjects (Fn, n = 1-4) were randomly chosen as single family unit excluding recipient, and mean values of 3D coordinates (Fx, Fy, Fz) at set landmark locations defined shape of family facial phenotype (F).
2. Displacement vector was calculated as the difference between 3D coordinate values at set landmark location of individual family member and corresponding mean family values and was called family identity index for that landmark. 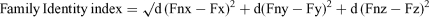
3. Mean and standard deviation values of identity indices (mm) of a particular landmark and nose length (mm) of all family members were indicated on a diagrammatic chart that depicted family identity pool (Fig 4).

### Facial allograft transplantation and identity changes prediction

**Figure 5:**
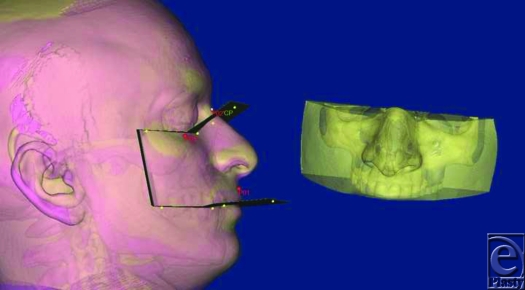
Recipient and donor's images were superimposed and using cutting plane (CP), appropriate sized graft was cut out.

**Figure 6:**
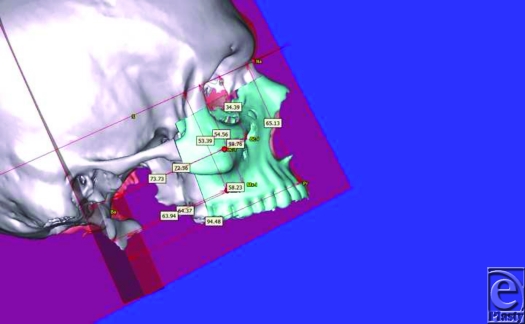
Three-dimensional measurements of recipient's craniofacial complex after facial allotransplantation.

Using point registration (Mimics version 14.0) at infraorbital margin, nasion, and prosthion on facial skeleton, recipient and donor's transparent facial images were superimposed; donor graft in the mid face area was cut out using a cutting plane that included lower half of nasal bone, whole maxilla including most of the hard palate and zygoma; and was transplanted onto recipient's face (Fig 5). Four subjects received facial allograft from 2 donors that resulted in 8 facial allotransplants. Identity indices were calculated for defined landmark locations of recipient's changed identity (Fig 6) and native identity and plotted on biophysical identity range chart. If they fell within identity range, it depicted identity transfer: otherwise, facial allotransplantation resulted in a new identity.

In addition, a donor compatible with family phenotype was chosen, and recipient's family identity indices were plotted on chart. If they fell within identity pool, recipient's new identity became part of family phenotype (Fig 4).

### Statistical analysis

All processes were performed by the author (A.M.). Errors in landmark localization were evaluated by comparing differences between 3D coordinates and linear measurements of original and repeated examinations of 10 subjects during a 2-week time interval. Method error was calculated as 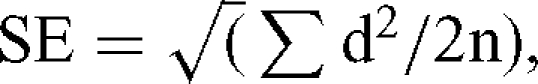 where “d” is the difference between double measurements and “n” is the number of paired double measurements.

## RESULTS

Table 2 shows 3D coordinate values of landmark locations and identity indices (identical twins only), and Table 3 shows 3D coordinate values and identity indices between new identity resulting from facial allotransplant and native identity, while Table 4 shows values of shape of family phenotype and family identity pool. Measurement errors of intraobserver precision were 1.1 mm, 1.0 mm, 1.3 mm for the x, y, and z coordinates, respectively; it was 1.4 mm for linear measurements. No statistical difference was detected between original and repeated measurements.

## DISCUSSION

To study variations among human faces, it is necessary to study the structures that compose it—skull characteristics, musculature, and associated soft tissue. The mimetic musculature is stretched across facial skeleton like a mask and to a great extent; any change in form of facial bones causes variations in facial appearance.^8^ Facial allotransplantation is a type of composite tissue transplantation that replaces the missing anatomical structures with identical ones and differs from other organ transplantation in degree hence, composition of its anatomical elements. Accordingly, facial allotransplantation can be divided into 2 main categories: (1) myocutaneous: contain only soft tissues and (2) osteomyocutaneous: ingrain hard tissues as well. This study attempts to objectively address the identity issues of both categories in detail.

It is important to know the fundamentals of shape perception in 3 dimensions to understand identity variations of human face. Perception of shape depends upon viewing direction, distance, and illumination. Shapes look different in 3 dimensions depending upon how much light is reflected on their surface and illumination of adjacent environment.^9^ If we assume an object of particular size that is made up of various small components, change in size and contours of any one component will not only reflect different amount of light but will also change the illumination of adjacent optical environment. Thus, object as a whole entity will be perceived different from its original shape. Similarly, human facial skeleton is made up of components whose individual size and shape is genetically predetermined, but their spatial arrangement is influenced by environmental factors^10^,^11^ and together, they lend human face a unique identity.

I hypothesize that craniofacial complex has 3 different optical environments that are contained by different size and contours of its skeletal components and, overlying soft tissues mask them. These three environments are inner, middle, and outer. Maxilla forms inner optical environment and is central to human identity. It not only supports the nose and lip but also there exist varied eminences, and angles within its small surface, along with overlying soft tissues, contribute to unique facial appearance. Its immediate outer boundaries form middle optical environment that is marked by landmarks buccal, prosthion, and maxillare. Boundaries of outermost optical environment are ill-defined and depend upon face index and chin characteristics (Table 5). In addition, individual components of 3 environments have biophysical range of size variation within which they are perceived identical, and physical parameters that define this range are identity-compatible to each other.

Contours of a bony segment are a true expression of its total morphological configuration,^12^ while geometric morphometric is a structured approach to analyze landmarks for shape variations.^13^ Moreover, variations in facial morphology correspond to cranial base, acting as bridge between neurocranium and facial cranium; thus, any change in cranial base configuration is reflected on the face.^14^ Reference planes in this study were set in a way that measured the physical dimensions of contours of facial bones based on cranial base anatomy. Displacement vector was calculated for the difference between 3D coordinates of a particular landmark location of same twin individuals that was called identity index. Identity indices defined the limit of biophysical variations in size of middle optical environment.

**Figure 7:**
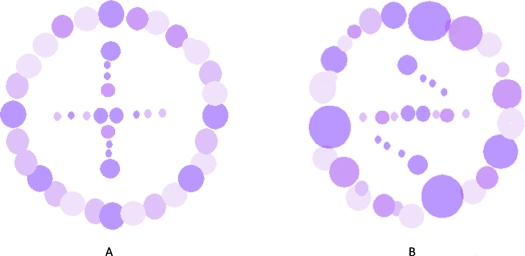
Inner environment of an object (*A*) will interact optically with the changed outer environment and hence, will be perceived different (*B*), even with no change in configuration of its morphology.

**Figure 8:**
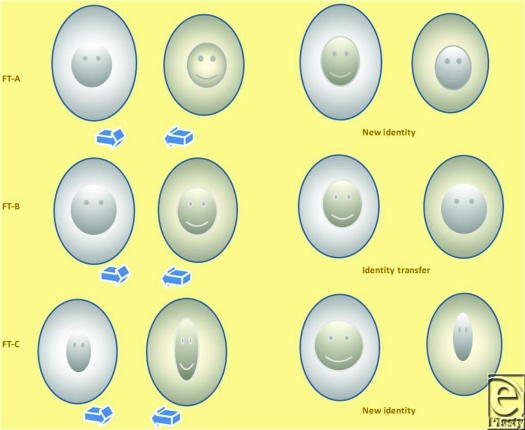
Two subjects of different morphological configurations. Large circle represents size of outer optical environment while small circle depicts size and morphology of middle and inner optical environments, respectively. Identity transfer occurs (FT-B) when outer and middle environments are compatible in shape and size; otherwise, it results in new identity.

To study a shape, one needs a constant stimulus that illuminates it.^15^ Thus, if size of components in the immediate optical environment of an object is kept constant, its shape can be distinctly appreciated and will be perceived different with any change in the environment (Fig 7). Similarly, if geometry of outer and size of middle optical environments of recipients and donors lie within a biophysical range, donor's face will be distinctly appreciated when transplanted on the recipient's face, and recipient's appearance will closely resemble the donor. In addition, if a donor's nose characteristics and maxillary shape are same, recipient's native identity will be restored to an extent. But if they are different, a donor's identity will be distinctly noticed on a recipient's face. This explains the phenomenon of identity transfer in facial allotransplantation. The same stands true in myocutaneous type of facial allotransplantation if craniofacial characteristics of donor and recipient are same; donor's myocutaneous facial mask will resume its native identity on recipient's facial skeleton. It proves wrong the common belief that no identity transfer occurs in facial allotransplant patients.^16^,^17^ Nonetheless, if there is a mismatch between outer and middle optical environments of recipient and donor, when transplanted, donor graft will optically interact with newly changed environment and whole craniofacial complex will be perceived as a new identity (Fig 8).

**Figure 9:**
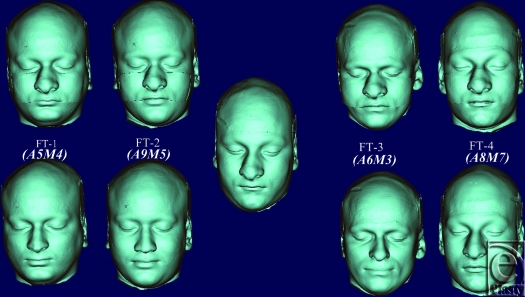
Representative of corresponding new facial images (*top*) of recipients (*bottom*) resulting from facial allotransplantation. Identity scores are shown in the parenthesis.

**Figure 11:**
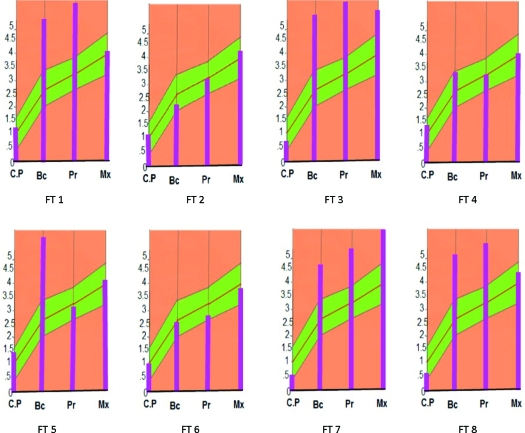
Identity indices (mm) of recipients plotted on biophysical identity chart indicating if facial allotransplantation resulted in identity transfer.

**Figure 12:**
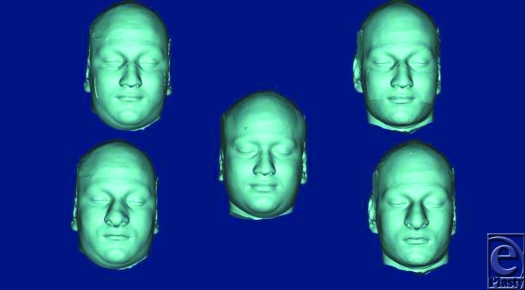
Representative of facial allotransplantation of identical twins of different face index and its effect on identity.

Donor's facial graft will retain its native identity only when recipient's parameters of outer optical environments match with the donor's. Face index indicates the overall shape of craniofacial complex, and average face index ranges from 85% to 89.99 %.^18^ In FT-4, recipient and donor are identical twins, but their face index is out of normal range (Fig 9 and Table 2. See also Fig 12). Parameters of their middle optical environment are in identity range, and it is noticed that recipient's identity has been nearly restored moreover; recipient resembles the donor to an extent. Mandibular chin has considerable effect in determining facial appearance,^19^ and facial graft will look same only when recipient's chin shape characteristics are compatible with the donor's. In diagrammatic charts (Fig 11), CP is chin protuberance difference between donor and recipient. Effects of chin shape on identity transfer can be clearly seen in FT-1, FT-2, FT-5, and FT-6. Higher body mass index will not only alter the face index but also affect optical perception of inner environments and hence, any possible identity changes. Identity transfer can be clearly seen in FT-2 and FT-6 (Figs 9 and 12) because the size of outer optical environments is in identity range. Morphological incompatible recipient and donor (FT-3, FT-7, and FT-8) will result in new identity.

**Figure 13:**
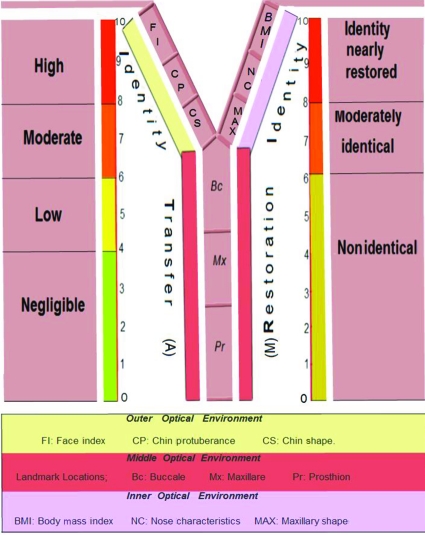
“AM” scale that quantifies the identity changes of facial allotransplantation. Left and right limb of scale quantifies the identity transfer (A) and identity restoration (M), respectively.

Aesthetic outcome of the facial allotransplantation will depend upon number of craniofacial parameters that are identity-compatible between recipient and donor. Scoring them will make easy to communicate identity issues with donor's family and recipient as well. “AM” scale, denoting letter “M,” is proposed in this study to quantify identity changes of both types of facial allotransplantation (Fig 13). Lee and Freire^20^ found that change in configuration of facial features affects the perception of geometric shape that comes in visual field of face and significantly distorts its overall perception. Moreover, vertical dimension has a considerable effect on face perception. On the basis of these attributes, highest score of 2 was given to identity-compatible parameters of outer and inner optical environment, while parameters of middle optical environment got highest score of 1, except Pr received the score of 2, considering the significance of vertical dimension. All identity incompatible parameters scored 0 (Table 6). Therefore, cross face transplantation of identical twins of same body mass index results in high identity transfer (A-10), and recipient's native identity is nearly restored (M-10). In addition to other aesthetic parameters such as gender, skin color, and texture, minimum morphometric parameters should be used to find a near identical match. Two types of parameters are proposed: primary parameters are nose characteristics and maxillary shape while secondary parameters are x coordinates of Bc and Mx.

Using this morphometric method and values of biophysical variations, identity transfer to recipient can be objectively predicted and quantified from donor's face CT scan. A drawback of this study is the small number of subjects. There needs collaboration among institutions to establish a twin research study with large sample size that measures and standardize the numerical values of biophysical variations in morphology of craniofacial components.

Humans use facial comparisons to recognize their relatives and regulate their behavior.^21^ Therefore, facial allotransplantation recipients will have to cope with reactions of family and friends to their altered appearance.^22^ In addition, family members share certain facial characteristics that make a unique family phenotype. Although ideal aesthetic goal of full facial allotransplantation would be to nearly restore the recipient's identity, it appears as a hypothetical proposition when preinjury CT scan is not available to ascertain the anatomy of recipient's true identity. If a recipient's new identity resembles to his/her family members, it will be a psychological relief and relatively easy to adapt to social reactions. In addition, this will give recipient's family a moral support that is already under stress because of their loved one's radical surgery. Morphometric method proposed in this study will help chose a donor graft to give recipient an identity that can be a part of family phenotype. To achieve this objective, a family facial skeletogram for morphometric studies should be planned to find a family identical donor. Recently, cone beam CT has been found more useful tool for dentofacial imaging because of reduced radiation exposure and favorable cost-benefit analysis.^23^ Moreover, facial allograft donation may be encouraged if a donor's family knows that their dead relative's graft will not be recognized; instead, it will become a part of another family phenotype.

This study can also be helpful (1) for organ banks to record the identity databases of facial grafts that can be used in future to choose appropriate graft in accordance with recipient's identity expectations from facial allotransplantation, (2) to measure and restore the facial symmetry of subjects that require treatment with facial implants for facial asymmetry, trauma, and congenital deformities, and (3) in twin studies, to accurately measure the inheritance patterns of craniofacial components keeping in mind the limitations of 2D methods.^23^

Severe facial disfigurement disrupts ability to recognize oneself and may constitute a major life crisis with a potential change in personality traits.^24^ This may lead to psychological disturbances such as reclusiveness, depression, anxiety, and suicidality, among others.^25^ Moreover, ability to cope with such a situation is subjective that depends upon self-esteem^26^ and accordingly, certain patients may have unrealistic expectations from facial allotransplantation.^27^ Pomahac et al^6^ predicted negligible appearance transfer through image editing of human color photographs. It is a subjective perception-based study that does not take into account the importance of third dimension which is essential to overall form and shape of an object. It is akin to a popular survey that records common opinion about morphological characteristics of a circular shape that in reality is a spherical object. If there is an identity transfer that patient did not expect, it will lead to an identity split, consequences of which would be grave if the recipient failed to psychologically accept the organ and could not rebuild its social expression in everyday life.^3^,^28^ Example of first hand transplant recipient further supports the aforementioned speculation who stopped taking immune-suppressive drugs considering the grafted hand as “other's” and subsequently, his hand was amputated.^29^ If similar situation arises with a facial allograft recipient, loss of the facial allograft will leave the patient in a worse condition before the surgery.^30^ Above all, patient may feel that he was coerced being into having this innovative surgery.^22^ Recently, US Department of Defense awarded research grant of $ 3.4 million to Brigham and Women's hospital, to perform facial allotransplantation on injured soldiers in Afghanistan and Iraq war.^31^ Keeping in mind, America's vast biological diversity, probability of identity transfer could be even higher.

## Acknowledgments

The author thanks his family, especially his beloved Mom, Mrs Urmila Devi (JBT, BA, and BEdu), for her indispensable spiritual inspiration and support that was a guiding force to finish this study. He also thanks his uncle, Mr Vijay Garg (superintendent of education, Department of Higher Education, Dist. Una, HP, India), who was instrumental in arranging the twins. Lastly, he thanks to Materialise team at Michigan, United States, particularly Eric Renteria, for the technical assistance regarding software.

## Figures and Tables

**Figure 10:**
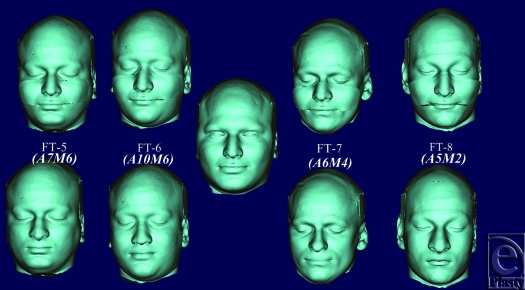
Representative of corresponding facial images (*top*) of recipients (*bottom*) resulting from face transplantation. Identity scores are shown in the parenthesis.

**Table 1:** Landmarks used in this study

Landmark	Abbreviation	Definition
Nasion	Na	Most posterior point on curvature between frontal bone and nasal bone in the mid sagittal plane
Basion	Ba	Most anterior point of foramen magnum
Sella	S	Geometric center of the pituitary fossa
Buccale	Bc	Most prominent point on external surface of each zygomatic arch, where arch turns medially
Maxillare	Mx	Point of maximum concavity on contour of maxilla directly below the lower contour of maxillozygomatic process
Prosthion	Pr	Point of maxillary alveolar process between left and right maxillary incisors
Menton	Me	Lowest border of the midmandibular suture
Pogonion	Pg	Most anterior midpoint of symphysis of mandible
Point B	B	Most posterior point of bony curvature of mandible above Pg
Chin protuberance	CP	Perpendicular distance from the Pg to B-Me line
Zygion	Zp	Most lateral point on the outline of each zygomatic arch
Face index	FI	(Na-Me distance)/(distance between right and left Zp) 100%.

**Table 2:** Values (mm) of x, y, and z coordinates of landmark locations, identity indices, and other morphological parameters*

		T1A	T1B	T2A	T2B	T3A	T3B	T4A	T4B	Mean	SD	T5A†	T5B†
Bc-X		102.93	105.37	97.8	98.27	110.31	109.29	100.03	97.14			98.51	100.83
	dx	2.44		0.47		1.02		2.89				2.32	
Bc-y		69.3	67.18	73.72	72.25	66.97	64.92	74.3	75.45			73.71	71.49
	dy	2.12		1.47		2.05		1.15				2.22	
Bc-z		29.6	28.62	29.32	28.99	27.96	27.03	29.69	29.75			32.15	35.59
	dz	0.98		0.33		0.93		0.06				3.44	
II		3.33		1.57		2.25		3.10		2.6	0.8		
Mx-X		58.9	59.04	61.01	60.75	61.03	59.57	64.11	62.06			58.33	62.02
	dx	0.14		0.26		1.46		2.05				3.69	
Mx-Y		64.24	60.46	61.38	62.49	62.95	60.59	64.76	63.96			63.7	65.35
	dy	3.78		1.11		2.36		0.8				1.65	
Mx-z		47.29	48.52	52.68	54.87	52.76	51.2	51.84	49.71			51.58	51.81
	dz	1.23		2.19		1.56		2.13				0.23	
I.I		3.97		2.45		3.18		3.06		3.2	0.6		
Pr-y		94.8	92.55	95.49	96.98	91.44	89.54	95.41	92.44			94.26	98.03
	dy	2.25		1.49		1.9		2.97				3.87	
Pr-z		57.74	60.59	66.43	64.35	61.6	62.65	68.86	70.06			62.53	61.68
	dz	2.85		2.08		1.05		1.2				0.95	
II		3.63		2.55		2.17		3.2		2.9	0.7		
CP		3.75	2.2	3.1	2.84	2.6	2.9	1.45	2.97			3.65	5.51
Δ		1.55		0.26		0.3		1.52		0.9	0.7	1.96	
BMI		19.9	18.6	25.2	25.3	17.1	19.9	22.3	23.7			23.3	21.5
FI		85	86	89	86	85	83	80	89.8			89	85
NL		50.56	48.37	47.86	47.67	53.63	51.94	51.94	50.03			39.51	47.03
Δ		2.19		0.19		1.69		1.91		1.49	0.89		

*Δ indicates difference; BMI, body mass index; CP, chin protuberance; FI, face index; II, identity indices; SD indicates standard deviation.

†Nonidentical twins.

**Table 3:** Values (mm) of x, y, and z coordinates of landmark locations, Identity Indices of transplanted faces – (FT n, n = 1-8)*

		FT 1(T5B-t4b†)	FT 2(T5A- t4b†)	FT 3(T1A- t4b†)	FT 4(T4A- t4b†)	FT 5(T5B-t2a†)	FT 6(T5A- t2a†)	FT 7(T1A-t2a†)	FT 8(T4A- t2a*)
Bc-X		97.14	97.14	97.14	97.14	97.8	97.8	97.8	97.8
	dx‡	3.69	1.37	5.79	2.89	3.03	0.71	5.13	2.23
Bc-y		74.22	72.72	71.28	74.81	75.08	73.63	69.2	77.65
	dy‡	2.73	0.99	1.98	0.51	3.59	0.08	0.1	3.35
Bc-z		32.79	32.2	31.71	30.83	29.18	29.84	27.75	26.29
	dz‡	2.8	0.05	2.11	1.14	6.41	2.31	1.85	3.4
II		5.37	1.43	6.47	3.3	7.49	2.41	5.45	5.26
Mx-X		62.06	62.06	62.06	62.06	61.01	61.01	61.01	61.01
	dx‡	0.04	3.73	3.16	2.05	0.01	2.68	2.11	3.1
Mx-Y		67.19	63.94	64.14	67.05	66.16	63.38	59.91	65.64
	dy‡	1.74	0.24	0.1	2.29	0.81	0.32	4.33	0.88
Mx-z		54.8	52.27	52.16	51.56	54.3	52.46	52.18	50.14
	dz‡	2.99	0.69	4.87	0.28	2.49	0.88	4.89	1.7
II		3.45	3.79	5.88	3.07	2.61	2.83	7	3.62
Pr-y		92.28	95.59	93.57	96.07	99	95.62	92.3	97.4
	dy‡	5.85	1.33	1.23	0.66	0.97	1.36	2.5	1.99
Pr-z		66.51	64.31	66.2	65.97	64.69	64.57	63.88	63.47
	dz‡	4.83	2.18	8.46	2.89	3.01	2.04	6.14	5.39
II		7.58	2.5	8.54	2.96	3.15	2.41	6.62	5.74
C.P		5.51	3.65	3.75	1.45	5.51	3.65	3.75	1.45
	Δ	1.54	1.68	0.78	1.52	2.41	0.55	0.65	1.65

*Δ indicates CP difference between donor and recipient; CP, chin protuberance; II, identity index.

†Bold and small letters indicate recipient and donor, respectively.

‡Coordinate value difference between recipient's new and native identity.

**Table 4:** Values (mm) of x, y, and z coordinates of landmark locations, identity indices, and other parameters used in family morphometric study*

		F1	F2	F3	F4	Mean	SD	FP†	FT‡	D§
Bc-X		102.93	105.37	97.8	98.27			101.1	100.03	100.03
	dx∥	2.9	5.07	3.5	2.03				0.98	
Bc-y		69.3	67.18	73.72	72.25			70.6	64.92	74.3
	dy∥	0.76	2.88	3.66	2.19				5.68	
Bc-z		29.6	28.62	29.32	28.99			29.1	27.03	29.69
	dz∥	0.5	0.48	0.22	0.11				2.07	
II		3.03	5.85	5.66	2.96	4.4	1.6		6.12	
Mx-X		58.9	59.04	61.01	60.75			59.9	64.11	64.11
	dx∥	1	0.86	1.11	0.85				4.21	
Mx-Y		64.24	60.46	61.38	62.49			62.1	61.03	64.76
	dy∥	0.14	1.64	0.72	0.39				1.07	
Mx-z		47.29	48.52	52.68	54.87			50.8	51.2	51.84
	dz∥	3.51	2.28	1.88	4.07				1.4	
II		2.13	2.8	2.29	4.1	2.8	0.9		4.62	
Pr-y		94.8	92.55	95.49	96.98			95	90.02	95.41
	dx∥	0.2	2.45	0.49	1.98				4.98	
Pr-z		57.74	62.59	66.43	63.35			62.5	66.02	68.86
	dy∥	2.76	0.09	3.93	0.85				3.52	
II		2.76	2.45	3.95	2.15	2.8	0.8		6.09	
NL		50.56	45.37	53.03	51.94			50.2	49.75	49.8
		0.36	4.83	2.83	1.74	2.4	1.9		0.45	

*II indicates identity index; NL, nose length; SD, standard deviation.

†Shape of family phenotype.

‡Recipient's new identity.

§Donor.

∥Coordinate value difference between new and native identity.

**Table 5:** Different optical environments of face and their components

Inner optical environment	• Nose characteristics–(a) nose length, (b) nose shape.
(Central to facial identity)	• Maxillary shape.
	• Body mass Index–affects soft tissue thickness.
Middle optical environment	• Buccal (Bc)–signifies malar eminences.
(Geometric bridge between outer and inner environments)	• Maxillare (Mx)–signifies mid-face width.
	• Prosthion (Pr)–signifies length of mid-face.
Outer optical environment	• Face index (FI)–defines overall geometry of cranio-facial complex.
(Defines overall shape of craniofacial complex)	• Chin characteristics–(a) chin protuberance, (b) chin shape.

**Table 6:** Show identity scores and following parameters was used to define identity compatibility

Optical Environments	Craniofacial Parameters		Identity Compatible	Identity Incompatible
	1. Nose	Shape	1	0
Inner		Lengthβ	1	0
	2. Maxillary shape		2	0
	3. Body mass index		2	0
	1. Bcβ		1	0
Middle	2. Pr β		2	0
	3. Mxβ		1	0
	1. Face index		2	0
Outer	2. Chin shape		2	0
	3. Chin protuberanceβ		2	0

Nose shape: bulbous, flat and straight nose

Chin shape: round and bi-fid chin

Body mass index: underweight, normal weight, overweight and obese categories

Face index: Normal–85%-89.99 %)

Maxillary shape: triangular and semilunar

β: biophysical range values.
